# Supplementary material for: The mediating effect of triglycerides and related indices on the association between polycyclic aromatic hydrocarbons and oral health in adults aged ≥ 45 years from the national health and nutrition examination survey 2003–2016
Source: Lipids Health Dis. 2026 Jan 5;25:35. doi: 10.1186/s12944-025-02847-5 (PMC12870046; doi:10.1186/s12944-025-02847-5)
Supplement: Supplementary file 1 — Supplementary material 1. [file 12944_2025_2847_MOESM1_ESM.docx]

**Supplementary Material**

Table S1. Comparison of characteristics between final analytical participants and excluded participants

| Variable | Final participants  (n = 4,442) | Excluded participants  (n = 17,534) | P value |
| --- | --- | --- | --- |
| Age, years (median [IQR]) | 62 (52, 71) | 62 (53, 73) | <0.001 |
| Sex |  |  | 0.080 |
| Female | 2213 (49.8) | 8996 (51.3) |  |
| Male | 2229 (50.2) | 8538 (48.7) |  |
| Race |  |  | <0.001 |
| Hispanic | 963 (21.7) | 4090 (23.3) |  |
| Non-Hispanic White | 2332 (52.5) | 8174 (46.6) |  |
| Others | 1147 (25.8) | 5270 (30.1) |  |
| Education |  |  | <0.001 |
| High | 1183 (49.4) | 4143 (45.5) |  |
| Low | 1210 (50.6) | 4953 (54.5) |  |
| Marital status |  |  | <0.001 |
| Married | 2604 (58.6) | 9794 (55.9) |  |
| Others | 1838 (41.4) | 7740 (44.1) |  |
| Poverty status |  |  | <0.001 |
| High | 3261 (73.4) | 10839 (70) |  |
| Low | 1181 (26.6) | 4640 (30) |  |
| BMI |  |  | 0.066 |
| <25 kg/m^2^ | 1108 (24.9) | 4268 (26.3) |  |
| ≥25 kg/m^2^ | 3334 (75.1) | 11944 (73.7) |  |
| Smoking |  |  | 0.573 |
| No | 3666 (82.5) | 14386 (82.2) |  |
| Yes | 776 (17.5) | 3125 (17.8) |  |
| Alcohol consumption |  |  | <0.001 |
| No | 1707 (38.4) | 6292 (42.4) |  |
| Yes | 2735 (61.6) | 8555 (57.6) |  |

IQR, interquartile range

Table S2. Sensitivity analysis using stricter case definitions for oral health problems

| Metabolite | Cases/Total | OR (95% CI)^*^ | P-value |
| --- | --- | --- | --- |
| 1-NAP | 434/4442 | 1.128 (1.021-1.246) | 0.020 |
| 2-NAP | 434/4442 | 1.230 (1.028-1.471) | 0.026 |
| 3-FLU | 434/4442 | 1.185 (1.000-1.405) | 0.053 |
| 2-FLU | 434/4442 | 1.043 (0.861-1.263) | 0.668 |
| 1-OHP | 434/4442 | 1.180 (1.017-1.368) | 0.031 |

1-NAP, 1-Hydroxynaphthalene. 2-NAP, 2-Hydroxynaphthalene. 3-FLU, 3-Hydroxyfluorene. 2-FLU, 2-Hydroxyfluorene. 1-PHE, 1-Hydroxyphenanthrene. 1-OHP, 1-Hydroxypyrene. OR, odds ratios .95%CI, 95% confidence intervals

^*^ Adjusted: adjusted for sex, age, race, education, marital status, BMI, smoking status, alcohol consumption, poverty status, DII, and UA.


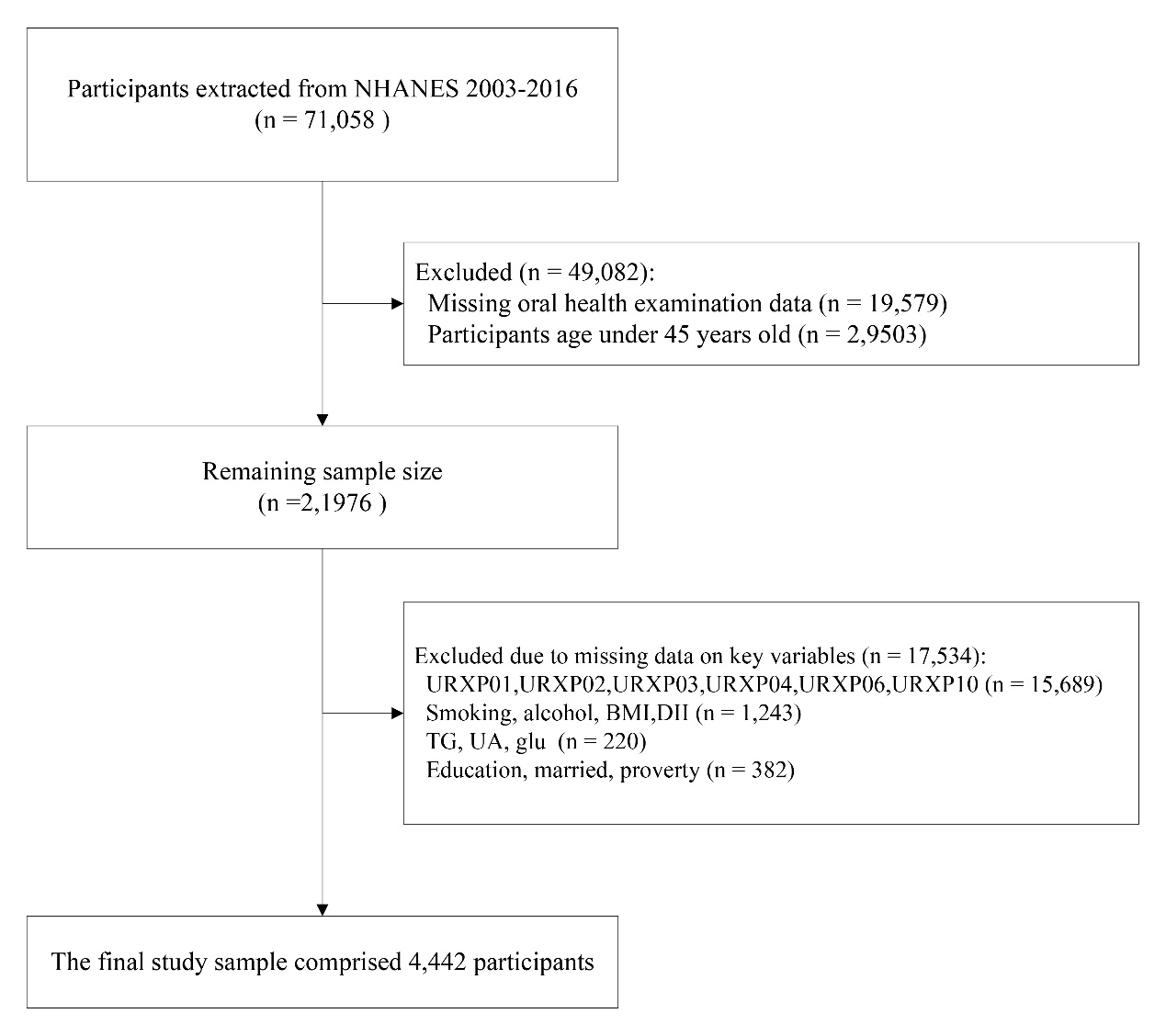


Figure S1. Flowchart of participant selection from the National Health and Nutrition Examination Survey (NHANES), 2003–2016.


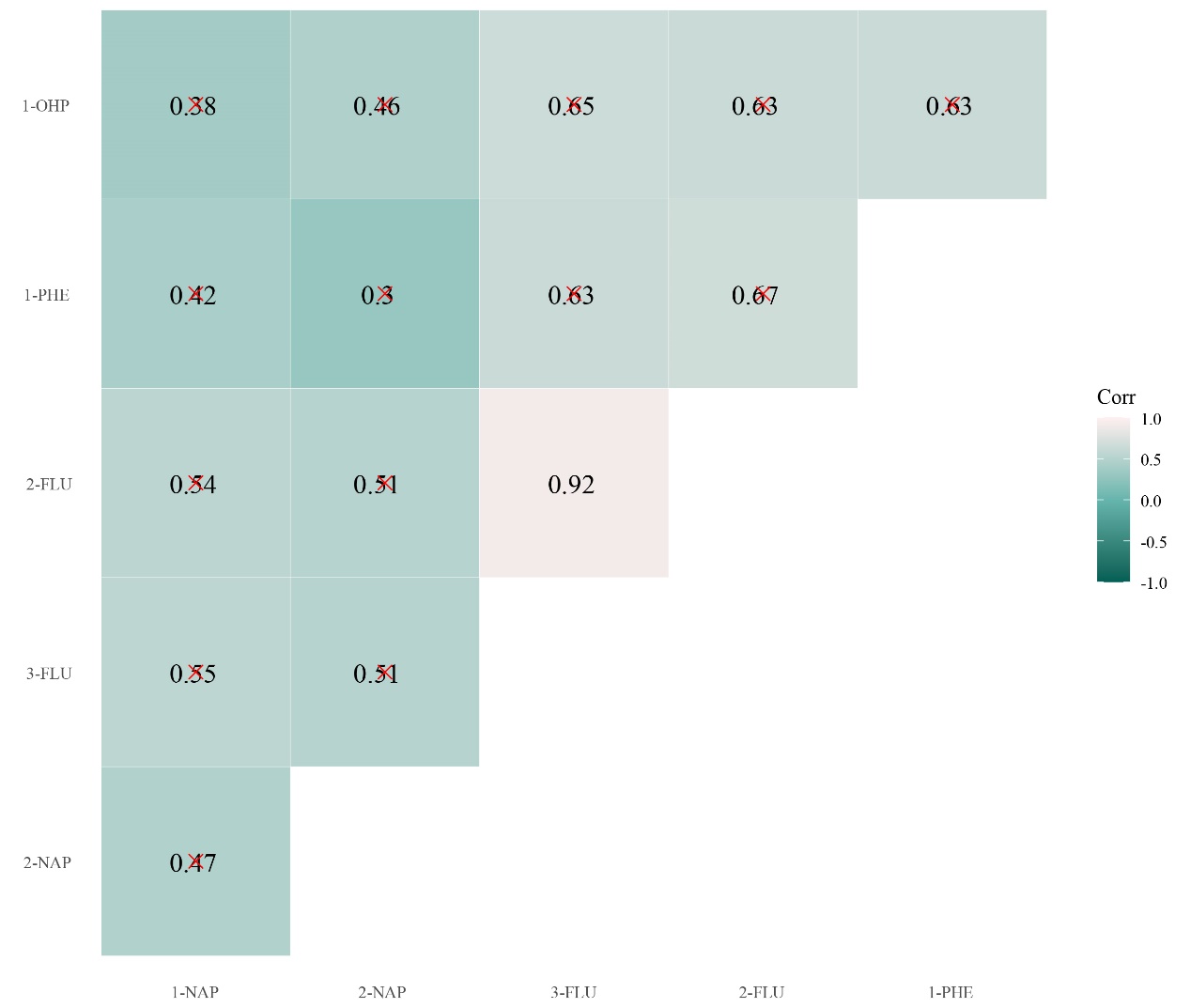


Figure S2. Spearman correlation analysis of urinary polycyclic aromatic hydrocarbon (PAH) metabolites. 1-NAP, 1-Hydroxynaphthalene. 2-NAP, 2-Hydroxynaphthalene. 3-FLU, 3-Hydroxyfluorene. 2-FLU, 2-Hydroxyfluorene. 1-PHE, 1-Hydroxyphenanthrene. 1-OHP, 1-Hydroxypyrene.


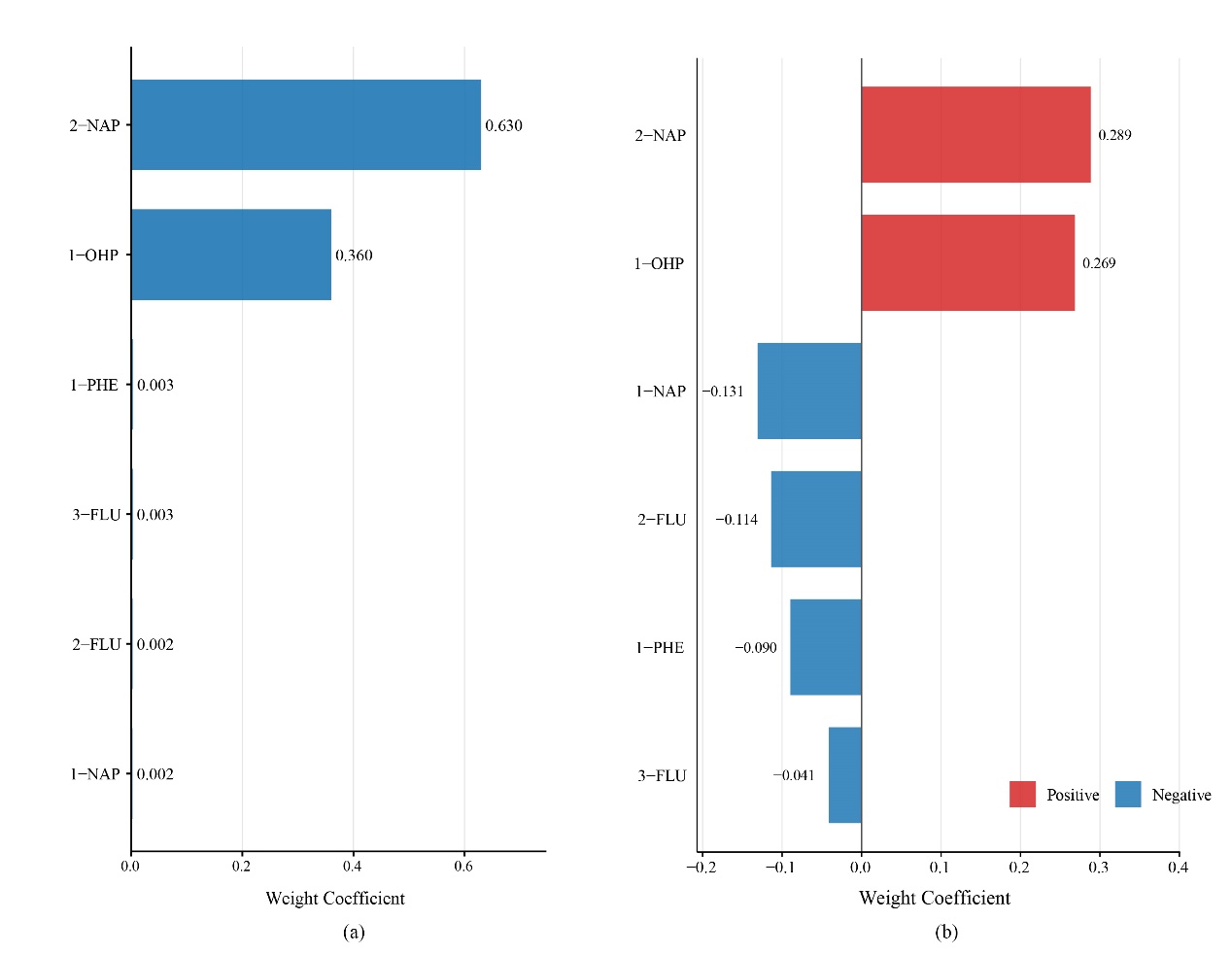


Figure S3. Estimated weights of individual polycyclic aromatic hydrocarbon (PAH) metabolites in mixtures associated with oral health. (a) weighted quantile sum (WQS) regression, (b) quantile-based g-computation (QGC) models. 1-NAP, 1-Hydroxynaphthalene. 2-NAP, 2-Hydroxynaphthalene. 3-FLU, 3-Hydroxyfluorene. 2-FLU, 2-Hydroxyfluorene. 1-PHE, 1-Hydroxyphenanthrene. 1-OHP, 1-Hydroxypyrene.


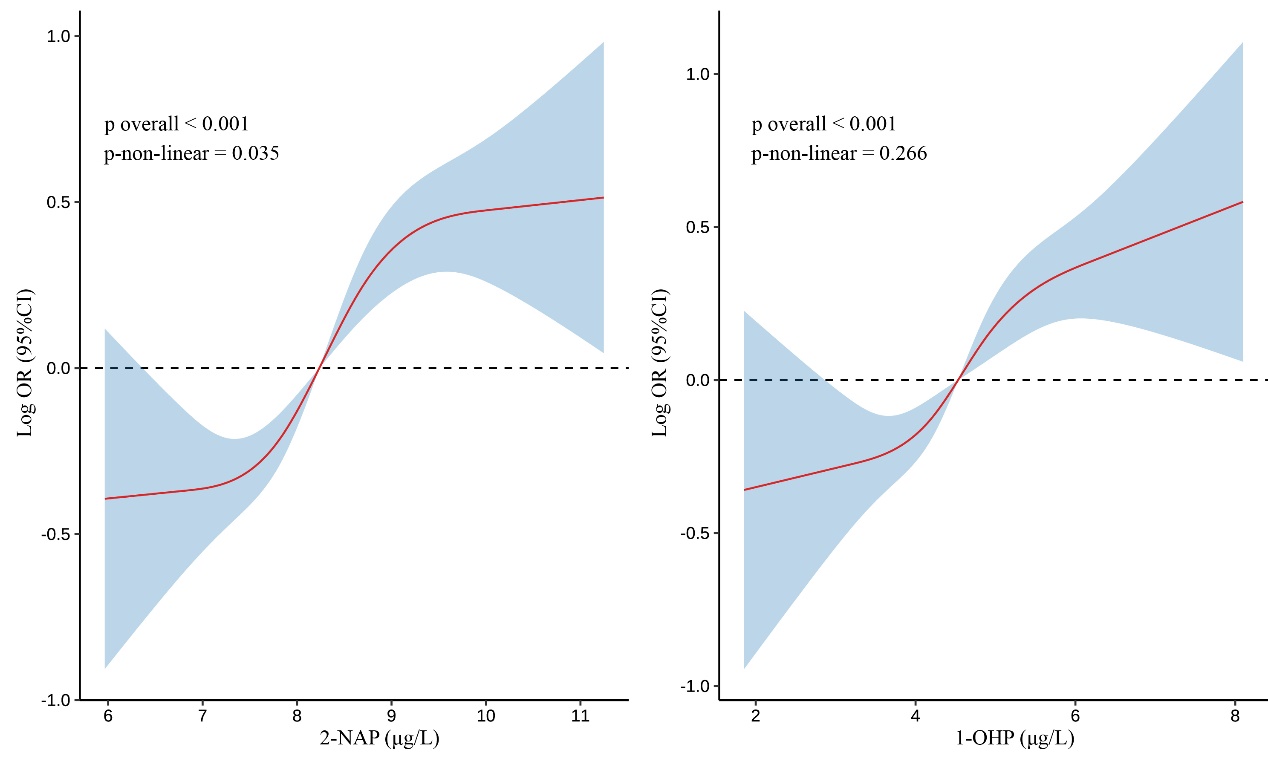


Figure S4. Exposure‒response associations of urinary 2-NAP and 1-OHP concentrations with oral health outcomes using restricted cubic splines. 2-NAP, 2-Hydroxynaphthalene. 1-OHP, 1-Hydroxypyrene.
