# Supplementary material for: The mediating effect of triglycerides and related indices on the association between polycyclic aromatic hydrocarbons and oral health in adults aged ≥ 45 years from the national health and nutrition examination survey 2003–2016
Source: Lipids Health Dis. 2026 Jan 5;25:35. doi: 10.1186/s12944-025-02847-5 (PMC12870046; doi:10.1186/s12944-025-02847-5)
Supplement: Supplementary file 2 — Supplementary material 2. [file 12944_2025_2847_MOESM2_ESM.pdf]

Editing Certificate

This document certifies that the manuscript

The mediating effect of triglycerides and related indices on the association between polycyclic aromatic hydrocarbons and oral health in adults aged ≥45 years from NHANES 2003–2016

prepared by the authors

Hua Shui, Weiling Liu, Qujie Li, Junhao Zhang, Cifeng Gao, Yong Wu, Chong Zeng, Wuling Chen, Fei Ma, Weiqi Liu

was edited for proper English language, grammar, punctuation, spelling, and overall style by one or more of the highly qualified English speaking editors at AJE.

This certificate was issued on **December 18, 2025** and may be verified on the AJE website using the verification code **E679-4AAB-095A-CA92-777F**.

Neither the research content nor the authors' intentions were altered in any way during the editing process. Documents receiving this certification should be English-ready for publication; however, the author has the ability to accept or reject our suggestions and changes. To verify the final AJE edited version, please visit our verification page at aje.com/certificate.
If you have any questions or concerns about this edited document, please contact AJE at support@aje.com.

AJE provides a range of editing, translation, and manuscript services for researchers and publishers around the world.
For more information about our company, services, and partner discounts, please visit aje.com.
