# Supplementary material for: The mediating effect of triglycerides and related indices on the association between polycyclic aromatic hydrocarbons and oral health in adults aged ≥ 45 years from the national health and nutrition examination survey 2003–2016
Source: Lipids Health Dis. 2026 Jan 5;25:35. doi: 10.1186/s12944-025-02847-5 (PMC12870046; doi:10.1186/s12944-025-02847-5)
Supplement: Supplementary file 3 — Supplementary material 3. [file 12944_2025_2847_MOESM3_ESM.pdf]

iThenticate

The mediating effect of triglycerides and related indices on the association between polycyclic aromatic hydrocarbons and oral health in adults aged ≥45 y...

Dec 20, 2025

6360 words / 37822 characters

The mediating effect of triglycerides and related indices on the ...

Sources Overview

13%

OVERALL SIMILARITY

#	Source	Type	Similarity
1	storage.googleapis.com	INTERNET	1%
2	www.frontiersin.org	INTERNET	1%
3	Miaomiao Jiang, Hui Zhao. "Joint association of heavy metals and polycyclic aromatic hydrocarbons exposure with depression in adu...	CROSSREF	<1%
4	public-pages-files-2025.frontiersin.org	INTERNET	<1%
5	bmcpublichealth.biomedcentral.com	INTERNET	<1%
6	Shaoqian Cui, Jiajun Wu, Xuekui Li, Xiaofei Zhang, Zonglong Nie, Yingjie Li, Wenzhi Xiang, Xiubo Jiang. "Association between polycycli...	CROSSREF	<1%
7	Preprint source<br>www.researchsquare.com	INTERNET	<1%
8	"Oral Immunology", Springer Science and Business Media LLC, 2026	CROSSREF	<1%
9	bmcgeriatr.biomedcentral.com	INTERNET	<1%
10	assets-eu.researchsquare.com	INTERNET	<1%
11	www.nature.com	INTERNET	<1%
12	www.ncbi.nlm.nih.gov	INTERNET	<1%
13	Qian Yang, Lingling Zeng, Jinfa Huang, Jianxiong Wuliu, Hai Liang, Kaixian Deng. "Association between urinary polycyclic aromatic hy...	CROSSREF	<1%
14	pure.manchester.ac.uk	INTERNET	<1%
15	Leigh F. Callahan, Jack H. Shreffler, Mary Altpeter, Britta Schoster et al. "Evaluation of group and self-directed formats of the Arthritis F...	CROSSREF	<1%
16	aacrjournals.org	INTERNET	<1%

17 Yibo Li, Yuhao Liu, Tao Yin, Mi He, Changyun Fang, Xiong Tang, Shifang Peng, Yundong Liu. "Association of periodontitis, tooth loss, a... <1%
CROSSREF

18 pesquisa1.bvsalud.org <1%
INTERNET

19 Xiaowei Zang, Wei Zhou, Hengguo Zhang, Xiaodong Zang. "Using Four Machine Learning Methods to Analyze the Association Betwee... <1%
CROSSREF

20 lipidworld.biomedcentral.com <1%
INTERNET

21 Amber L. Cathey, Deborah J. Watkins, Zaira Y. Rosario, Carmen M. Vélez Vega et al. "Polycyclic aromatic hydrocarbon exposure result... <1%
CROSSREF

22 Jinru Liu, Wenqian Zheng, Hongchen Sun, Fermín E. González, Ding Zhou, Xiaowei Xu. "Environment-adaptive microneedle design stra... <1%
CROSSREF

23 Kelly K. Ferguson, Thomas F. McElrath, Gerry G. Pace, David Weller et al. "Urinary Polycyclic Aromatic Hydrocarbon Metabolite Associ... <1%
CROSSREF

24 breast-cancer-research.biomedcentral.com <1%
INTERNET

25 www.mdpi.com <1%
INTERNET

26 www.researchgate.net <1%
INTERNET

27 Danielle Gallegos, Naiyana Wattanapenpaiboon, Mark L. Wahlqvist. "Food and Nutrition - Sustainable Food and Health Systems", Routl... <1%
PUBLICATION

28 Deborah Marshall, Rodrigo Dal Ben, Gillian Currie, Rae Yeung et al. "How Does Juvenile Idiopathic Arthritis Affect the Work and Usual ... <1%
CROSSREF

Preprint source
29 Marziyeh Shafizadeh, Vikram Bhatia, Samah Ahmed, Britt Drögemöller et al. "Bitter taste genetics and oral health in Canadian Longitu... <1%
CROSSREF POSTED CONTENT

30 Sneha S. Patil, Naveen Puttaswamy, Ajay Pillarisetti, Andres Cardenas et al. "Association of prenatal and early life polycyclic aromatic ... <1%
CROSSREF

31 Vittorio Dibello, Silvano Quarto, Frank Lobbezoo, Karl G. H. Parisius et al. "Associations among self-reported oral health and intrinsic c... <1%
CROSSREF

32 Wood, Lisa G, Nitin Shivappa, Bronwyn S Berthon, Peter G Gibson, and James R Hebert. "Dietary inflammatory index is related to asth... <1%
CROSSREF

33 Yizhao Huang, Hongling Zhang, Yiqing Lv, Ling Yu, Hongxiu Liu, Shunqing Xu, Tian Chen, Yuanyuan Li. "Joint association of polycyclic ... <1%
CROSSREF

34 ifnmujournal.com <1%
INTERNET

35 link.springer.com <1%
INTERNET

36 www.jkaoms.org <1%
INTERNET

37 www.scielo.br <1%
INTERNET

38 ChunXiang Bao, Jie Luo, ShuYing Miao. "Association of Urinary Metabolites of Polycyclic Aromatic Hydrocarbons with Urinary Inconti... <1%
CROSSREF

39 Mengying Xi, Qijun Ruan, Sulan Zhong, Jiatong Li, Weijuan Qi, Congman Xie, Xiaoyan Wang, Nuerbiya Abuduxiku, Jia Ni. "Periodontal b... <1%
CROSSREF

40 Ron Stout, Daniel Reichert, Rebecca Kelly. "Lifestyle Medicine and the Primary Care Provider - A Practical Guide to Enabling Whole Per... <1%
PUBLICATION

41 Saeed Asgary, Alireza Akbarzadeh Baghban, Fatemeh Mahmoudi Afsah. "Global Burden of Dental Caries and Oral Disorders: A 31-Yea... <1%
CROSSREF

42 Shin'ichi Okamoto, Nguyen Thi Kim Oanh. "Statistics in Environmental Monitoring and Assessment", CRC Press, 2026 <1%
PUBLICATION

43 Xianghong Zhou, Kun Jin, Shi Qiu, Qiuxiang Yang, Peng Wang, Yu Zhan, Xinyi Huang, Zhongyuan Jiang, Dan Hu, Lu Yang, Qiang Wei. "A... <1%
CROSSREF

44 Xiaoxia Liu, Xiuli Zhao, Lu Ye, Chengfeng Hu, Zhihao Xie, Jianan Ma, Xia Wang, Wei Liang. "The TyG Index Mediates Air-Pollution-Asso... <1%
CROSSREF

45 Yating Wang, Enwei Zhu, Xiaoyan Zhu, Xiaoliang Li, Mei'an He, Rihong Zhai, Xuli Wu, Dongsheng Hu, Xu Han. "Exposure to polycyclic ar... <1%
CROSSREF

Excluded search repositories:

- None

Excluded from document:

- Bibliography

Excluded sources:

- None

Excluded preprints

- None

The mediating effect of triglycerides and related indices on the association between polycyclic aromatic hydrocarbons and oral health in adults aged ≥45 years from NHANES 2003–2016

Abstract

Background: Environmental pollutants are known determinants of oral health; however, the specific effects of polycyclic aromatic hydrocarbons (PAHs) on the oral health of adults aged ≥ 45 years remain poorly understood. This study investigated the association between PAH exposure and oral health and examined the mediating roles of triglyceride (TG) levels, the Triglyceride–Glucose index (TyG), and the Triglyceride–Glucose–Body Mass Index (TyG-BMI).

Methods: This study utilized data of 4,442 individuals from the National Health and Nutrition Examination Survey. Logistic regression, weighted quantile sum (WQS) regression, and quantile-based g-computation (QGC) analyses assessed associations of urinary PAH metabolites with self-reported poor oral health. Mediation analysis evaluated the roles of TG, TyG, and TyG-BMI.

Results: Exposure in the highest quartile to 2-naphthol (2-NAP) and 1-hydroxypyrene (1-OHP) was associated with poor oral health, with odds ratios (95% confidence intervals) of 1.96 (1.34–2.87) and 2.26 (1.51–3.39), respectively. The WQS and QGC models confirmed the overall positive effect of PAH mixtures, driven mainly by 2-NAP and 1-OHP. Mediation analysis revealed that TG, TyG, and TyG-BMI significantly mediated 4.13 to 4.87% of the effect of 2-NAP, whereas TyG-BMI mediated 6.10% of the effect of 1-OHP. A significant interaction between 2-NAP and race was observed in the subgroup analysis

Conclusions: Exposure to specific PAHs is linked to poor oral health and is partially

mediated by triglyceride-related pathways. These results underscore the critical need to reducing PAH exposure and control high triglyceride levels to improve oral health outcomes and promote health equity among middle-aged and older adults, providing a scientific basis for enhancing healthy ageing and fostering healthier living environments.

Keywords

Body mass index; Mediation analysis; Oral health; Polycyclic aromatic hydrocarbons; Triglyceride

1. Introduction

Oral health a major global public health challenge, with World Health Organization (WHO) estimates suggesting that around 3.69 billion people worldwide are affected by oral diseases [1]. Studies indicate a higher prevalence of oral diseases among those aged ≥45 years. Ageing is accompanied by diminished reparative potential of periodontal apparatus and age-related immunosenescence [2], and immune function weakens, making this population more susceptible to pathogenic factors [3]. The WHO reports indicate that 42% of the population in the Western Pacific Region was affected by oral diseases in 2023, with people aged 45 and older comprising more than 60% of the cases. Additional studies suggest that oral health awareness is generally lower among people in this age group [4], and research conducted in Switzerland among participants aged ≥45 years demonstrated a significant link between age and caries and/or periodontal disease [5]. Similarly, a survey of Canadian adults aged 45 to 85 years found that over 30% of participants described multiple oral health concerns over the previous 12

months [6].

Furthermore, oral diseases exhibit strong linkages with several systemic disorders, including cardiovascular ailments, and deficits in nutritional metabolism and immune competence [7-9]. Therefore, further research into the epidemiological characteristics, pathogenic mechanisms, and comprehensive prevention and treatment strategies for oral diseases in people aged 45 and above is urgently needed.

The development of oral diseases is affected by multiple factors, including genetics, behaviour, inflammation, and environmental pollution [10-13]. Among these, the involvement of polycyclic aromatic hydrocarbons (PAHs) as potential risk factors has garnered increasing attention. PAHs belong to a category of environmentally persistent chemical pollutants defined by their condensed aromatic ring systems [14] and are widely present in both the natural environment and environments influenced by anthropogenic activities. Major exposure sources include household cooking, coal combustion, industrial emissions, and vehicular exhaust [15]. Numerous studies have shown that the accumulation of PAHs in the body can contribute to the pathological processes of various diseases, and their potential harm to oral health is being increasingly recognised [16, 17]. Current evidence supporting a link between PAHs and oral conditions is still insufficient. For instance, the underlying mechanisms through which PAHs affect oral health remain unclear, and differences in susceptibility to PAH exposure among various populations, particularly in middle-aged groups such as those aged 45 years, have not been sufficiently studied.

In addition, while triglycerides (TGs) and related indices, such as the Triglyceride–Glucose Index (TyG) and the Triglyceride–Glucose–Body Mass Index (TyG-BMI), have been widely demonstrated to play mediating roles in metabolic diseases [18-20], their capacity to act as mediators in the relationship between PAH exposure and oral

diseases remains unclear. Elucidating the role of TG-related indicators in this context is especially critical for understanding health risks in the population aged 45 years, highlighting the importance of further investigation into these mechanisms for developing targeted prevention strategies.

To address this gap, we evaluated the link between PAH exposure and oral health in adults aged ≥45 years and further assessed whether TG, TyG, and TyG-BMI mediate this association. We hypothesised that exposure to specific PAH correlates with adverse oral health outcomes and that this association is partially mediated through triglyceride-related metabolic pathways. Clarifying these underlying mechanisms is vital to elucidate how pollutant exposure mediates oral diseases in this population, which could inform focused interventions.

2. Methods

2.1 Study cohort

This study analysed data from seven cycles of the NHANES, covering the period from 2003 to 2016. All selected cycles contained complete information on exposure, outcome, and covariate variables, ensuring the data integrity and reliability of the analyses. The study initially included 71,058 participants. After excluding individuals aged under 45 years and those with missing covariate data, the final analytical cohort comprised 4,442 subjects. A detailed participant selection flowchart (Figure S1) illustrates the exclusion criteria and the number of individuals excluded at each stage. A comparison was performed to examine possible selection bias between the included participants (n = 4,442) and eligible individuals aged ≥45 years who were excluded owing to missing data on covariates (n = 17,534). A comparison of demographic and socioeconomic characteristics revealed that the groups were broadly similar. Importantly, no significant differences were observed in key health-related variables, including sex, BMI, or

smoking status, supporting the representativeness of the final cohort for the analyses (Table S1). The study received ethical approval from the National Center for Health Statistics Ethics Review Board (Protocols #98-12, #2005-06, and #2011-17). All participants provided written informed consent.

2.2 Assessment of Urinary PAH Metabolites

In this study, internal PAH exposure was assessed using urinary concentrations of monohydroxy-PAH metabolites from the NHANES database as biomarkers. We concentrated on six specific metabolites: 1-naphthol (1-NAP), 2-naphthol (2-NAP), 3-hydroxyfluorene (3-FLU), 2-hydroxyfluorene (2-FLU), 1-hydroxyphenanthrene (1-PHE), and 1-hydroxypyrene (1-OHP). Analyses were conducted via isotope-dilution on an integrated system for online purification, chromatographic separation, and tandem mass spectrometry, following enzymatic deconjugation of urine samples prior to automated extraction and chromatographic separation. The method demonstrated robust sensitivity, with detection limits of 0.008 – 0.09 ng/mL, as supported by linear calibration curves and stringent quality control measures. This validated methodology provided reliable quantification of exposure biomarkers for investigating associated health outcomes. Urinary concentrations were creatinine (Cr)-adjusted to account for variations in urine dilution, despite the physiological determinants of Cr excretion [21, 22], this correction was applied to align the data with the NHANES methodology. The normalised concentrations (in ng/g Cr) were subsequently natural log-transformed to better meet the assumptions of the parametric statistical models.

2.3 Outcome Measures

This study principally assessed oral health status as a binary variable (good vs. poor). Given the use of self-reported questionnaire data rather than clinical diagnoses, the construct of "poor oral health" was operationalised as a state characterised by a patient-

perceived negative impact on their quality of life or a negative global self-assessment. This approach is methodologically aligned with established public health research, which similarly defined health status groups by collapsing self-reported ratings (e.g., “fair/poor”) to capture meaningful negative perceptions of health [23]. During the 2003–2008 survey cycle, the variable OHQ630 ("How often do you feel bad because of your mouth?") was used. Participants who reported responses such as “very often”, “fairly often”, or “occasionally" were considered to have experienced a perceptible negative bearing of oral health on their daily living and were thus assigned to the disease group. For the 2009–2016 survey cycle, the variable OHQ845 ("Overall, how would you rate the health of your teeth and gums?") was used as an alternative measure. Participants who rated their health as “fair” or “poor” were included in the disease group, reflecting a negative perception of their oral health. The control group included participants from their respective survey cycles who gave an affirmative response to either of the following criteria: reporting “hardly ever” or “never” to OHQ630 or rating their oral health as “excellent”, “very good”, or “good” on OHQ845. Individuals with either negative self-perception or impaired quality of life were collectively defined as the disease group (i.e., poor oral health), thereby capturing a population with a clinically meaningful burden of symptoms for analysis.

2.4 Covariates

On the basis of previous studies [24-26] and data from this study population, we selected the following covariates: sex, age, race, education, marital status, body mass index (BMI), smoking status, alcohol consumption, poverty status, dietary inflammatory index (DII), and biochemical indicators (TG, uric acid [UA], and glucose [GLU]).

Age was categorised into three groups: <60 years, 60–74 years, and ≥75 years.

Race was classified as Hispanic, non-Hispanic White, or other (which included non-Hispanic Black, multiracial and other individuals). Socioeconomic variables included education level (low: high school or below; high: college or above), poverty status (low: family income-to-poverty ratio [PIR] <1.3; high: PIR ≥1.3), and marital status, categorised as married or other (including never married, divorced, or widowed). BMI was categorised as <25 kg/m^2 or ≥25 kg/m^2. The lifestyle factors assessed included smoking status, categorised as non-smoker (comprising never and former smokers) or current smoker, and alcohol consumption status, classified as nondrinker (including never and former drinkers) or current drinker. The DII is a measure of a diet's overall inflammatory potential [27], and the score was derived from NHANES dietary data and dichotomised as low (DII ≤ 0) or high (DII > 0). Serum concentrations of TG and UA were measured using the timed endpoint method, and the GLU concentration was determined by the hexokinase method with sample blank correction.

2.5 Statistical Analysis

In accordance with the complex survey design of NHANES, a weighting methodology was applied in this study. Analytical guidelines for NHANES were followed, which included appropriate adjustment of the individual cycle weights to incorporate the seven cycles of data under examination. Continuous variables are summarised as medians with interquartile ranges, and categorical variables are reported as unweighted counts alongside weighted proportions. To avoid distributional assumptions and to ensure the robustness of statistical inference, nonparametric tests were employed for intergroup comparisons, and differences between the control and disease groups were assessed using chi-square tests. The associations among the PAH metabolites were assessed using Spearman's rank correlation coefficient. Both univariate and multivariate logistic regression models were subsequently employed to evaluate the associations between

these metabolites and the prevalence of oral health conditions. The findings are reported as odds ratios (ORs) alongside their 95% confidence intervals (CIs). The multivariate models were adjusted for potential confounders, including sex, age, race, education, marital status, BMI, smoking status, alcohol consumption, poverty status, DII, and UA. To account for multiple comparisons inherent in the quartile-based analysis of each PAH metabolite, the false discovery rate (FDR) was controlled via the Benjamini–Hochberg procedure, implemented separately for each metabolite.

To address the frequent high correlations among environmental exposures, our analysis utilised two complementary statistical methods: weighted quantile sum (WQS) regression and quantile-based g-computation (QGC). In the WQS approach, the six PAH metabolites were quartiles and combined into a weighted index. Stable estimates of component weights and their confidence intervals were derived from 1000 bootstrap samples. The resulting weights reflect the relative contribution of each metabolite to the overall mixture effect. As a complementary method, QGC was used to estimate the combined effect on the outcome following a simultaneous one-quartile increase in all exposures, thereby capturing both positive and negative contributions within the mixture. Both models were adjusted for the following covariates: sex, age, race, education, marital status, BMI, smoking status, alcohol consumption, poverty status, DII, and UA. For each model, the weights of individual PAH metabolites were calculated and visualised using bar plots to illustrate the relative importance of the mixture components.Subsequently, we performed restricted cubic spline (RCS) analysis on PAH metabolites that were significant in logistic regression and consistently identified as predictors in both WQS and QGC models.

To further investigate potential mechanistic pathways, a mediation analysis was performed using key PAH metabolites identified in previous analyses, testing the hypothesis that their effects may be partially mediated by triglycerides and related

indices (TyG and TyG-BMI). The natural direct effect (NDE), natural indirect effect (NIE), and total effect were estimated, with 95% confidence intervals for the mediation effects based on 1000 bootstrap resamples. A significant mediation effect was defined as a confidence interval excluding zero. The TyG index was calculated as ln[TG×FBG/2], and the TyG-BMI index was calculated as TyG×BMI. Finally, subgroup analyses were performed: interaction terms between key PAH metabolites and these stratification variables were included in multivariate models to evaluate potential effect modification.

To test the sensitivity of the results to case definitions, a sensitivity analysis was conducted using stricter criteria for both survey cycles. For the 2003–2008 cycle, the case group was redefined to include only participants reporting “very often” or “fairly often” to OHQ630, excluding those reporting “occasionally”. For the 2009–2016 cycle, the case group was redefined to include only those reporting “poor” on OHQ845, excluding those reporting “fair”. All primary multivariate logistic regression analyses of these PAH metabolites were repeated using these more stringent case definitions.

R software was employed for all statistical analyses, using a two-sided $P < 0.05$ as the criterion for statistical significance.

3. Results

This study included a total of 4,442 participants, who were categorised into a disease group (n = 1,121) and a control group (n = 3,321) on the basis of oral health status. Between the two groups, significant differences were noted in multiple variables, with the disease group showing different distributions regarding age, race, education, marital status, smoking status, poverty status, DII, TG, GLU, and PAH metabolites—including 1-NAP, 2-NAP, 3-FLU, 2-FLU, and 1-OHP (all $P < 0.05$). In contrast, there were no significant differences in sex, BMI, alcohol consumption, or Cr, UA, or 1-PHE levels

(all $P > 0.05$) (Table 1). a significant increasing trend in the prevalence of oral diseases (P for trend < 0.001), which was consistent across all sex and race subgroups (Table 2).

As shown in Figure S2, the Spearman correlation analysis demonstrated statistically significant positive correlations for a number of urinary PAH metabolites (all $P < 0.05$), with particularly strong correlations observed between 2-FLU and 3-FLU ($\rho = 0.92$), between 2-FLU and 1-PHE ($\rho = 0.67$), and between 3-FLU and 1-PHE ($\rho = 0.63$). These findings suggest potential coexposure patterns or common sources, which may lead to combined effects on oral health. To systematically evaluate the associations, logistic regression models were used to assess the individual effects of each PAH metabolite on oral health status. Subsequently, WQS regression and QGC models were employed to quantify the combined effect of the mixture.

After covariate adjustment, we observed a significant increasing trend for both 2-NAP and 1-OHP quartiles and poorer oral health (P for trend < 0.001), with the highest quartiles showing ORs of 1.96 (95% CI: 1.34–2.87) and 2.26 (95% CI: 1.51–3.39), respectively. No significant associations were observed for 1-PHE, 1-NAP, 3-FLU, or 2-FLU (Table 3).

Both the WQS regression and QGC models, adjusted for covariates, demonstrated that exposure to the PAH mixture was positively linked to an increased risk of poor oral health. 2-NAP and 1-OHP were the most influential drivers of this adverse overall effect in both models (Figure S3). The results of the exposure–response relationship analysis for 2-NAP and 1-OHP are shown in Figure S4, revealing a nonlinear association for 2-NAP (P for nonlinear $= 0.035$).

The association between 2-NAP and 1-OHP with oral health was significantly mediated by TG and the TyG, with significant indirect effects and a proportional effect ranging from 4.13% to 4.87%. Furthermore, TyG-BMI served as a significant mediator in the

association between 2-NAP and oral health, with a proportion mediated of 6.10%. In contrast, the TyG-BMI did not significantly mediate the link between 1-OHP and oral health (Figure 1).

A significant interaction effect was observed for race on the association between 2-NAP and oral health (*P* for interaction = 0.017), with the strongest effect observed among Hispanic participants (OR = 1.497, 95% CI: 1.276–1.756). In contrast, no significant interaction effects were detected for any of the other subgroups in the association between 1-OHP and oral health (Figure 2).

Sensitivity analyses employing stricter case definitions (n = 434 cases) showed largely consistent association patterns linking urinary PAH metabolites to oral health problems, compared with the primary analyses. Specifically, significant positive associations persisted for 1-NAP, 2-NAP, and 1-OHP (OR = 1.128, 95% CI: 1.021–1.246; OR = 1.230, 95% CI: 1.028–1.471; and OR = 1.180, 95% CI: 1.017–1.368, respectively), whereas 3-FLU tended towards significance (OR = 1.185, 95% CI: 1.000–1.405; P = 0.053). The direction of these associations aligned with the exposure–response relationships observed in the primary analyses (Table S2).

4. Discussion

This study demonstrated significant associations between PAHs and poor oral health in adults aged ≥45 years. Both individual and mixture analyses consistently revealed that increased urinary concentrations of 2-NAP and 1-OHP were significantly linked to increased risk of oral diseases, with an exposure–response relationship observed for 2-NAP. Mediation analyses indicated that TG and TyG mediated these associations, whereas TyG-BMI partially mediated them. Furthermore, subgroup analyses revealed a significant effect modification by race on the association between 2-NAP and oral health. These findings emphasise the public health relevance of both individual and

combined PAH exposure as environmental determinants of the oral disease burden among adults aged 45 years and above.

Exposure to PAHs may negatively affect oral health through multiple mechanisms. Specifically, PAHs can initiate local inflammatory responses by activating aryl hydrocarbon receptors via their metabolites [28]. This activation enhances downstream inflammatory signalling and boosts the production of key pro-inflammatory cytokines [29-31]. These cytokines not only induce inflammation of the oral mucosa but also damage periodontal tissues, resulting in clinical manifestations such as gingival redness, swelling, and bleeding, thereby contributing to the development of periodontitis [32]. Furthermore, PAHs may disrupt intercellular interactions by altering the expression and distribution of key junctional complexes, thereby compromising the barrier function of the oral epithelium [33]. Consequently, PAHs can disrupt the oxidative–antioxidative balance by generating reactive oxygen species, subsequently activating apoptosis-related proteins, inducing apoptosis in oral epithelial cells, and ultimately contributing to the pathogenesis of conditions such as periodontitis, laryngitis, and oral cancer [34-36]. Moreover, PAHs can contribute to oral disease by inducing epigenetic changes in the oral cavity. For instance, dibenzo [*def,p*] chrysene alters gene expression during the early stages of oral cancer by causing hypomethylation within an intron of fibroblast growth factor 3 and hypermethylation within an intron of vesicle-associated membrane protein 3, thereby participating in the early development of oral cancer [37].

This study revealed that exposure to 2-NAP and 1-OHP is significantly associated with the occurrence of oral diseases. These findings both align and contrast with those of previous research. PAHs have been demonstrated to increase periodontitis risk [17] and to cause oral mucosal injury, with potential utility as oral cancer biomarkers [38]. Furthermore, positive correlations between periodontitis and both 3-OHF and 2-OHF

have been reported [39]. These studies indicate that PAH exposure may adversely affect oral health by dysregulating inflammatory pathways and inflicting cellular damage via oxidative stress and DNA impairment.

Methodological variations distinguish this work from earlier reports. Unlike previous studies on the combined effects of heavy metals and PAHs on periodontitis [17], this analysis focused exclusively on PAHs. In contrast to a prior study that assessed PAH-related biological effects via DNA adduct measurement in oral cells using a limited sample size [38], this study utilised NHANES data, quantified urinary PAH metabolites, and included a larger participant cohort. Moreover, whereas earlier work examined only the independent associations of individual PAHs with periodontitis [39], this study is the first to apply WQS and QGC models to systematically quantify the overall oral health risk per quartile increase in a mixture of six PAHs, identifying 2-NAP and 1-OHP as the primary drivers. These discrepancies may stem from differences in sample size, study design, statistical approaches, and the specific PAH metabolites analysed.

The results of the mediation analysis conducted in this study revealed that lipid metabolism markers—specifically, TG and the TyG index (a surrogate indicator of insulin resistance)—significantly mediated the association between two typical PAH metabolites (2-NAP and 1-OHP) and oral health impairment, with mediation proportions ranging from 4.13% to 4.87%. Importantly, this study further revealed that different PAH monomers may influence health through distinct metabolic pathways: obesity-related insulin resistance, represented by TyG-BMI, significantly mediated the relationship between 2-NAP and oral health (proportion of 6.10%), whereas this effect was not observed in the pathway involving 1-OHP. These findings provide important mechanistic insight by suggesting that the impact of 2-NAP on oral health may be more

dependent on insulin resistance pathways exacerbated by obesity. These findings align with those of previous research, which revealed that obesity is a key driver of insulin resistance and chronic inflammation [40, 41] and is capable of exacerbating metabolic disturbances and adversely affecting periodontal tissues. In contrast, the effects of 1-OHP are mediated primarily by lipid metabolic dysfunction and insulin resistance (as represented by the baseline TyG index), which appears largely independent of obesity status. This divergence may stem from heterogeneity in pharmacokinetics, receptor-binding affinity, or activation of specific toxic pathways among different PAH monomers. Although numerous studies have separately established independent pathways linking PAH exposure to metabolic dysregulation via oxidative stress and inflammation [42, 43] and connecting metabolic dysregulation to oral diseases [40, 41, 44], the present study integrated these segments by identifying specific serum biomarkers that mediate the relationship between individual PAH metabolites and oral health outcomes, thereby providing novel evidence for a continuous toxicological pathway from exposure to clinical outcome: environmental exposure to PAHs is involved in the pathogenesis of oral diseases partly through mechanistically disrupting host lipid and glucose metabolism. Nevertheless, the temporal sequence inferred from these mediation models warrants further validation in longitudinal studies. Such research should be designed to include biomarkers of oxidative stress and inflammation, with the aim of elucidating the broader mechanistic cascade connecting PAH exposure to oral health.

This study revealed that race was a significant effect modifier in the association between 2-NAPs and oral health, with the strongest effect observed in the Hispanic population; However, we found no evidence of effect modification by any subgroup on the association between 1-OHP and oral health. These findings share both consistencies

and differences with those of previous research. First, these results validate and extend prior observations—existing studies have reported that the associations between PAH metabolites and health outcomes may be modified by racial factors [26]. This study similarly identified a significant racial interaction effect for 2-NAP in the context of oral health, further supporting the view that demographic characteristics can amplify the health effects of PAHs. More importantly, these findings reveal that the Hispanic population is particularly vulnerable to the oral health effects of 2-NAP, expanding the understanding of race-specific risk patterns. Second, the results for 1-OHP in this study are consistent with those of previous research: similar to its lack of interaction effects in association with psoriasis [45], this study also found no subgroup interactions between 1-OHP and oral health, collectively suggesting that 1-OHP may have broader rather than specific health effects.

To investigate the relationship between urinary PAH metabolite levels and oral health, a common analytical strategy used in environmental epidemiology was applied for handling complex exposures—converting continuous concentrations into quartiles. This approach was selected for two main reasons: first, to capture potential nonlinear dose–response relationships while avoiding the loss of statistical power that may result from incorrectly assuming a linear model; and second, because quartile-based analysis provides more intuitive and robust risk estimates, which are less sensitive to outliers and easier to interpret. Notably, this methodological choice aligns with several recent studies on PAH-related health effects. For instance, Wu et al. [17], in their investigation of the relationship between PAHs and periodontitis, categorised metabolite concentrations into quartiles and identified 2-NAP and 1-OHP as key drivers using WQS and QGC models. Similarly, Dai et al. [46], in their study on PAHs and immunosuppression, adopted quartile categorisation and applied mixed models such as

QGC to confirm the significant immunosuppressive effects of PAH exposure. Thus, this study follows a well-established analytical framework within this field, and the consistency of the results across different models further strengthens the reliability of the conclusions.

The observed link between PAH metabolites and oral health problems remained stable when different diagnostic thresholds were applied. This consistency strengthens confidence in the robustness of the relationship. These findings are consistent with those of earlier reports. A study by Wu et al. [47], utilising data from the NHANES 2009–2014, confirmed a positive association between six hydroxylated polycyclic aromatic hydrocarbons (OH-PAHs) and periodontitis. Notably, the significance of this positive association was maintained even after restricting the analysis to a subsample from 2011–2014, confirming the robustness of the link between OH-PAHs and periodontitis. The consistency between the findings of the two studies likely stems from a genuine and robust biological link between PAHs and oral health issues, an effect sufficiently strong to remain detectable across varying analytical conditions. Consequently, the present findings not only reinforce the reliability of the conclusions but also extend the scientific understanding of PAHs as significant environmental risk factors for periodontal disease.

5. Strength and limitations

This study is the first to elucidate the partial mediating role of triglyceride-related metabolic pathways in the link between exposure to specific PAH and poorer oral health among individuals aged 45 years and older, who have a high burden of oral diseases, thereby providing important clues for targeted prevention in high-risk populations. However, this study has several limitations. First, the categorisation of PAH metabolites into quartiles for analysis may increase the risk of multiple comparisons and false-

positive findings; Although FDR correction was applied, the quartile-based analysis is still susceptible to multiple comparison error and false positives; this should be considered in interpretation. Second, the use of self-reported oral health status, rather than clinical assessment, may introduce recall bias or outcome misclassification, which could affect the accuracy of the findings. Additionally, the short biological half-lives of PAH compounds limit the reliability of single urinary metabolite measurements as indicators of long-term exposure. Furthermore, the external validity of these findings is confined to middle-aged and older populations, given the enrollment criteria requiring participants to be at least 45 years of age. Although this focus is justified by the higher disease burden in this group, the relationships between PAH exposure, TG-related indices, and oral health may differ in younger populations because of variations in physiology, exposure patterns, and lifestyle factors. Finally, although the models were adjusted for a wide range of covariates, residual confounding from unmeasured factors may persist. Notably, more granular determinants of personal exposure to atmospheric contaminants could not be obtained, such as individual time-activity patterns, microenvironmental pollution levels in domestic or occupational settings, or the use of protective equipment. Additionally, other unmeasured factors, including genetic susceptibility, oral hygiene practices, and access to dental care, represent potential sources of residual confounding. Therefore, future studies should employ longitudinal designs, incorporate clinical oral health assessments, and utilise repeated biomarker measurements to better establish causality and improve the accuracy of exposure and outcome evaluation.

6. Conclusion

This study demonstrated that specific PAH metabolites (2-NAP and 1-OHP) are significantly associated with an elevated risk of oral diseases in adults aged ≥45 years.

This association is partly mediated by disruptions in lipid metabolism and the promotion of insulin resistance, with obesity exacerbating the adverse effects of 2-NAP. These findings have immediate clinical relevance. For patient care, clinicians should incorporate an assessment of PAH exposure risk into the evaluation of middle-aged and older adults, particularly those with obesity. This can be achieved by integrating targeted questions on environmental and occupational exposure histories into routine health assessments. Identifying high-risk individuals through this approach would enable earlier and more tailored oral health surveillance and preventive strategies. From a public health perspective, the results provide robust evidence for policies aimed at reducing environmental PAH exposure to improve population oral health.

References

1. GBD 2021 Oral Disorders Collaborators. Trends in the global, regional, and national burden of oral conditions from 1990 to 2021: a systematic analysis for the Global Burden of Disease Study 2021. *Lancet* 2025, 405(10482):897-910.
2. Tonetti MS, Bottenberg P, Conrads G, Eickholz P, Heasman P, Huysmans MC *et al*: Dental caries and periodontal diseases in the ageing population: call to action to protect and enhance oral health and well-being as an essential component of healthy ageing - Consensus report of group 4 of the joint EFP/ORCA workshop on the boundaries between caries and periodontal diseases. *J Clin Periodontol* 2017, 44 Suppl 18:S135-S144.
3. Ravi MB, Srinivas S, Swamy KNR, Anupama A, Roy A, Gowrav MP *et al*: Influence of Domestic Exposure to Polycyclic Aromatic Hydrocarbons on Women's Periconceptional Stage and Associated Risk of Oral Cleft in Offspring. *J Pharm Bioallied Sci* 2022, 14(Suppl 1):S589-S594.

4. Pawinska M, Kondrat A, Jamiolkowski J, Paszynska E: Dental Status and Oral Health Behaviors of Selected 45-74-Year-Old Men from Northeastern Poland. *Int J Environ Res Public Health* 2023, 20(11):6005.

5. Borg-Bartolo R, Roccuzzo A, Tennert C, Prasinou M, Jaggi M, Molinero-Mourelle P *et al*: A Cross-sectional Study Investigating the Oral Health Status of Adult and Elderly Swiss Community-Dwellers. *Oral Health Prev Dent* 2025, 23:67-75.

6. De Rubeis V, Jiang Y, de Groh M, Dufour L, Bronsard A, Morrison H *et al*: Oral Health Problems among Canadians Aged 45 to 85: Data from the Canadian Longitudinal Study on Aging Baseline Survey (2011–2015). In: *Int J Environ Res Public Health* 2023, 20(8):5533

7. Park JH, Leem GH, Kim JW, Song TJ: Persisting Chronic Periodontal Disease as a Risk Factor for Cardiovascular Disease: A Nationwide Population-Based Cohort Study. *J Clin Periodontol* 2025, 52(3):375-386.

8. Ghanem AS, Nagy AC: Oral health's role in diabetes risk: a cross-sectional study with sociodemographic and lifestyle insights. *Front Endocrinol (Lausanne)* 2024, 15:1342783.

9. Chan AKY, Tsang YC, Jiang CM, Leung KCM, Lo ECM, Chu CH: Diet, Nutrition, and Oral Health in Older Adults: A Review of the Literature. *Dent J (Basel)* 2023, 11(9):222.

10. Zhang Y, Bian C, Yu C, Zhu M, Weir MD, Xu HHK *et al*: Bidirectional association between oral diseases caused by plaque and the inflammatory bowel disease: A systematic review and meta-analysis. *Jpn Dent Sci Rev* 2025, 61:7-21.

11. Chapple IL, Bouchard P, Cagetti MG, Campus G, Carra MC, Cocco F *et al*: Interaction of lifestyle, behaviour or systemic diseases with dental caries and periodontal diseases: consensus report of group 2 of the joint EFP/ORCA workshop on the boundaries between caries and periodontal diseases. *J Clin Periodontol* 2017, 44 Suppl 18:S39-S51.

12. Jepsen S, Blanco J, Buchalla W, Carvalho JC, Dietrich T, Dorfer C *et al*: Prevention and control of dental caries and periodontal diseases at individual and population level: consensus report of group 3 of joint EFP/ORCA workshop on the boundaries between caries and periodontal diseases. *J Clin Periodontol* 2017, 44 Suppl 18:S85-S93.

13. Zhu L, Tang M, Cai Y, Wang P: Association between exposure to environmental pollutants and increased oral health risks, a comprehensive review. *Front Public Health* 2024, 12:1482991.

14. Ali N, Ismail IMI, Khoder M, Shamy M, Alghamdi M, Costa M *et al*: Polycyclic aromatic hydrocarbons (PAHs) in indoor dust samples from Cities of Jeddah and Kuwait: Levels, sources and non-dietary human exposure. *Sci Total Environ* 2016, 573:1607-1614.

15. White AJ, Bradshaw PT, Herring AH, Teitelbaum SL, Beyea J, Stellman SD *et al*: Exposure to multiple sources of polycyclic aromatic hydrocarbons and breast cancer incidence. *Environ Int* 2016, 89-90:185-192.

16. de Abreu M, Cruz AJS, Borges-Oliveira AC, Martins RC, Mattos FF: Perspectives on Social and Environmental Determinants of Oral Health. *Int J Environ Res Public Health* 2021, 18(24):13429.

17. Fan Z, Yue G, Yu D, Zhang M, Kitaura H: The Joint Impact of Heavy Metals and Polycyclic Aromatic Hydrocarbons on Periodontitis. *Int Dent J* 2025, 75(5):100879.
18. O'Donovan SD, Erdos B, Jacobs DM, Wanders AJ, Thomas EL, Bell JD *et al*: Quantifying the contribution of triglycerides to metabolic resilience through the mixed meal model. *iScience* 2022, 25(11):105206.
19. Low S, Pek S, Moh A, Ang K, Khoo J, Shao YM *et al*: Triglyceride-glucose index is prospectively associated with chronic kidney disease progression in Type 2 diabetes - mediation by pigment epithelium-derived factor. *Diab Vasc Dis Res* 2022, 19(4):14791641221113784.
20. Wang X, Liu J, Yu K, Huang Z, Liu H, Li X: Association between TyG-related parameters and NAFLD risk in Japanese non-obese population. *Sci Rep* 2025, 15(1):7119.
21. Worsfold M, Davie MW, Haddaway MJ: Age-related changes in body composition, hydroxyproline, and creatinine excretion in normal women. *Calcif Tissue Int* 1999, 64(1):40-44.
22. Ávila M, Mora Sánchez MG, Bernal Amador AS, Paniagua R: The Metabolism of Creatinine and Its Usefulness to Evaluate Kidney Function and Body Composition in Clinical Practice. *Biomolecules,* 2025,15(1):41.
23. Gandhi K, Lim E, Davis J, Chen JJ: Racial-ethnic disparities in self-reported health status among US adults adjusted for sociodemographics and multimorbidities, National Health and Nutrition Examination Survey 2011-2014. *Ethn Health* 2020, 25(1):65-78.

24. Natarajan P, Madanian S, Marshall S: Investigating the link between oral health conditions and systemic diseases: A cross-sectional analysis. *Sci Rep* 2025, 15(1):10476.

25. Aldosari M, Helmi M, Kennedy EN, Badamia R, Odani S, Agaku I *et al*: Depression, periodontitis, caries and missing teeth in the USA, NHANES 2009-2014. *Fam Med Community Health* 2020, 8(4):e000583

26. Ye C, Liu Y, He Z, Huang W, Chen G, Peng T *et al*: Urinary polycyclic aromatic hydrocarbon metabolites and hyperlipidemia: NHANES 2007-2016. *Lipids Health Dis* 2024, 23(1):160.

27. Shivappa N, Steck SE, Hurley TG, Hussey JR, Hebert JR: Designing and developing a literature-derived, population-based dietary inflammatory index. *Public Health Nutr* 2014, 17(8):1689-1696.

28. Holme JA, Vondracek J, Machala M, Lagadic-Gossmann D, Vogel CFA, Le Ferrec E *et al*: Lung cancer associated with combustion particles and fine particulate matter (PM(2.5)) - The roles of polycyclic aromatic hydrocarbons (PAHs) and the aryl hydrocarbon receptor (AhR). *Biochem Pharmacol* 2023, 216:115801.

29. Khan IU, Mukhtar H, Haqqi TM: Chemical carcinogens increase IL-1 alpha and IL-6 gene transcripts in human keratinocytes. *Exp Dermatol* 1993, 2(2):84-88.

30. Ye J, Zhu R, He X, Feng Y, Yang L, Zhu X *et al*: Association of plasma IL-6 and Hsp70 with HRV at different levels of PAHs metabolites. *PLoS One* 2014, 9(4):e92964.

31. Liu B, Zhao X, He H, Zhou L, Fan Y, Chai X *et al*: Exposure to urinary polycyclic aromatic hydrocarbon metabolites for the effect of lung function

among children and adolescents: Epidemiological study and mechanism exploration. *Ecotoxicol Environ Saf* 2025, 292:117990.

32. Castro SA, Collighan R, Lambert PA, Dias IH, Chauhan P, Bland CE *et al*: Porphyromonas gingivalis gingipains cause defective macrophage migration towards apoptotic cells and inhibit phagocytosis of primary apoptotic neutrophils. *Cell Death Dis* 2017, 8(3):e2644.

33. Groeger S, Meyle J: Oral Mucosal Epithelial Cells. *Front Immunol* 2019, 10:208.

34. Wang Y, Andrukhov O, Rausch-Fan X: Oxidative Stress and Antioxidant System in Periodontitis. *Front Physiol* 2017, 8:910.

35. Chen KM, Guttenplan JB, Zhang SM, Aliaga C, Cooper TK, Sun YW *et al*: Mechanisms of oral carcinogenesis induced by dibenzo[a,l]pyrene: an environmental pollutant and a tobacco smoke constituent. *Int J Cancer* 2013, 133(6):1300-1309.

36. Choi H, Kim CS: Polycyclic Aromatic Hydrocarbons from Fine Particulate Matter Induce Oxidative Stress and the Inflammatory Response in Human Vocal Fold Fibroblast Cells. *Oxid Med Cell Longev* 2021, 2021:5530390.

37. Sun YW, Chen KM, Imamura Kawasawa Y, Salzberg AC, Cooper TK, Caruso C *et al*: Hypomethylated Fgf3 is a potential biomarker for early detection of oral cancer in mice treated with the tobacco carcinogen dibenzo[def,p]chrysene. *PLoS One* 2017, 12(10):e0186873.

38. Chen KM, Sun YW, Krebs NM, Sun D, Krzeminski J, Reinhart L *et al*: Detection of DNA adducts derived from the tobacco carcinogens, benzo[a]pyrene and dibenzo[def,p]chrysene in human oral buccal cells. *Carcinogenesis* 2022, 43(8):746-753.

39. Lin F, Wang H, Wang X, Fang Y: Association between exposure to multiple polyaromatic hydrocarbons and periodontitis: findings from a cross-sectional study. *Environ Sci Pollut Res Int* 2023, 30(52):112611-112624.

40. Stanisic D, Jeremic N, Majumder S, Pushpakumar S, George A, Singh M *et al*: High Fat Diet Dysbiotic Mechanism of Decreased Gingival Blood Flow. *Front Physiol* 2021, 12:625780.

41. Ladeira LLC, Nascimento GG, Leite FRM, Alves-Costa S, Barbosa JMA, Alves CMC *et al*: Obesity, Insulin Resistance, Caries, and Periodontitis: Syndemic Framework. *Nutrients* 2023, 15(16):3512

42. Zhou S, Li X, Dai Y, Guo C, Peng R, Qin P *et al*: Association between polycyclic aromatic hydrocarbon exposure and blood lipid levels: the indirect effects of inflammation and oxidative stress. *Environ Sci Pollut Res Int* 2023, 30(59):123148-123163.

43. Ribiere C, Peyret P, Parisot N, Darcha C, Dechelotte PJ, Barnich N *et al*: Oral exposure to environmental pollutant benzo[a]pyrene impacts the intestinal epithelium and induces gut microbial shifts in murine model. *Sci Rep* 2016, 6:31027.

44. Lee HJ, Lee JW, Kim S, Kwon YJ: Comparison of the triglyceride glucose index and modified triglyceride glucose indices in assessing periodontitis in Korean adults. *J Periodontal Res* 2023, 58(3):503-510.

45. Li JH, Yan XN, Fu JY, Hu HY: Impact of urinary PAHs on psoriasis risk in U.S. adults: Insights from NHANES. *PLoS One* 2024, 19(12):e0314964.

46. Dai Y, Deng Q, Liu Q, Zhang L, Gan H, Pan X *et al*: Humoral immunosuppression of exposure to polycyclic aromatic hydrocarbons and the roles of oxidative stress and inflammation. *Environ Pollut* 2024, 347:123741.

47. Wu Y, Yang H, Jin W, Wu Y, Yu Y, Chen Q *et al*: Association between polycyclic aromatic hydrocarbons and periodontitis: Results from a large population-based study. *J Clin Periodontol* 2024, 51(4):441-451.
